# Therapeutic options to prevent recurrence of an aggressive aneurysmatic bone cyst of the cervical spine of a 16 year old boy - a case report

**DOI:** 10.1186/1754-9493-5-20

**Published:** 2011-08-26

**Authors:** Juliane Richter, Sven K Tschöke, Jens Gulow, Uwe Eichfeld, Magdalena Wojan, Georg von Salis-Soglio, Christoph E Heyde

**Affiliations:** 1 Department of Orthopaedic Surgery, University Hospital Leipzig, Liebigstr. 20, 04103 Leipzig, Germany; 2 Department of Abdominal, Transplant, Vascular and Thoracic Surgery, University Hospital Leipzig, Liebigstr. 20, 04103 Leipzig, Germany

## Abstract

The aneurysmatic bone cyst (ABC) is a benign primary bone tumour. If located in the cervical spine, its expansive growth and destructive behaviour may lead to instability and serious neurological impairment. We report a case of a 16-year-old boy with an aggressive ABC in the 7^th ^cervical vertebra. Computertomographic and magnetic resonance imaging revealed the envelopment of the left 7^th ^and 8^th ^spinal nerve along with the anterior displacement of the left vertebral artery. The interdisciplinary surgical strategy consisted of a partially incomplete cyst resection, subtotal spondylectomy with posterior screw-and-rod fixation from C6-Th1, iliac crest bone grafting and anterior plating from C6-Th1. With regard to the high rate of recurrence after incomplete resection published in the recent literature, the patient was postoperatively treated by megavoltage radiotherapy with a total dose of 30Gy (daily dose of 1.8 Gy for 3 weeks). The clinical and radiographic follow-up showed complete recovery of all neurologic impairments and no signs of tumour recurrence at 3, 6 and 12 months after surgery. This case highlights diverse treatment regimens and shall outline the challenge and the problems of the interdisciplinary decision-making in adolescents presenting with ABC in high-demanding anatomical regions.

## Backround

Aneurysmatic bone cysts (ABC) are described to be benign, but locally destructive bony lesions. Representing approximately 1% of all primary bone tumours, 10-30% of ABC cases involve the spine, with approximately 22% of these located in the cervical spine region [1,2]. Its incidence is found to be within the first two decades and predominantly in females [3].

To date, the etiology of ABCs remains uncertain. Possible explanations include a posttraumatic intralesional hematoma, whereupon ABCs may be classified into primary (idiopathic) and secondary ABC, either evolving from a bleeding into a preexisting traumatic, infectious or malignant lesion [4]. Respectively, the latter is capable to mask a malignancy, such as a teleangiectatic osteogenic sarcoma, thus enforcing the need for multiple biopsies in cases where clinical and radiographic findings are suspicious [4].

According to Enneking's classification, ABC may be subdivided into three developmental stages:

1. State of latency without progressive growth and the tendency to spontaneous remission.

2. Active state with progressive growth, but without extravagating anatomic borders.

3. Aggressive state with expansive and osteolytic growth, thereby extravagating diverse tissue layers.

Radical surgical debridement and complete excision of the cystic wall should be the primary therapeutic option. When complete excision of the cystic wall is achieved, the overall prognosis is good and tumour recurrence is highly unlikely. The risk of local recurrence, however, correlates well both with the preoperative state and the radicalness of resection [5]. Therefore, advanced and precise staging is required to choose the appropriate operative strategy.

In the following, we report our therapeutic regimen and surgical strategy in the case of a 16 year old boy who presented with an expansive growing bone cyst of the 7^th ^cervical vertebra (stage 3 according to Enneking).

## Case Report

A 16 year old patient presented with a 4-month history of cervical pain, which was accompanied by a progressive radicular pain of his left arm. Within this time period he remarked a slight swelling of soft tissue in the neck and a weakness of his left arm when performing weight training.

His previous medical history was without comorbidities or surgical interventions. His family history was clear of malignancies.

On clinical examination there was a soft tissue swelling of the left anterior and lateral region of the neck and atrophy of the left biceps muscle. Neurologic examination revealed a slight weakness (4 out of 5 according to the Janda classification) and numbness of the left forearm and hand.

**Figure 1:**
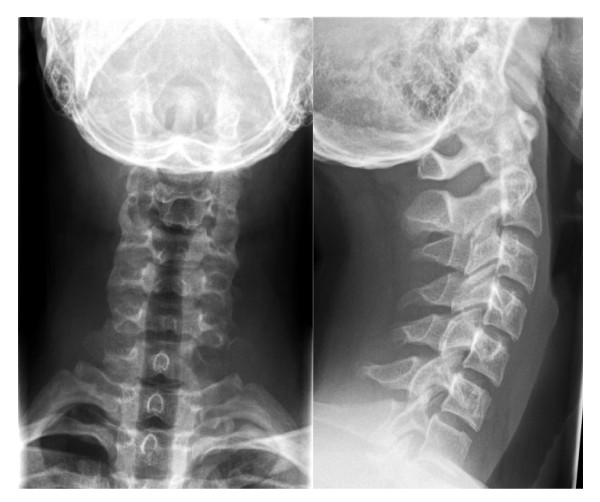
**The a.p. view of the preoperative conventional X-Ray of the cervical spine shows a thinning of bone in C7 on the left**.

**Figure 2:**
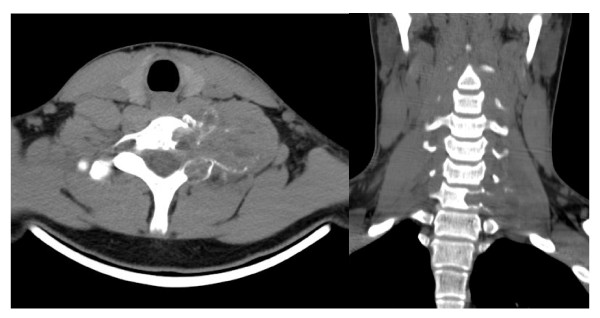
**The axial and coronal planes of the CT scan image show the localization and expansion of the bone cyst originating from C7**.

Conventional radiographs showed a clear osteolytic lesion of the 7^th ^cervical vertebra in both lateral and ap views (Figure 1). Consecutive MRI and CT scan verified an expansive bone cyst with partial osteolysis of the 7^th ^cervical vertebra, reaching far into the juxtavertebral tissues. Furthermore, the lesion comprised the 7^th ^and 8^th ^spinal nerve and revealed an anterior displacement of the left vertebral artery (Figure 2).

The histopathological finding of the CT guided biopsy confirmed an aneurysmatic bone cyst.

Given the expansive growth of the bony lesion and the neurological impairment, a primary and as radical as possible surgical debridement and excision was indicated.

**Figure 3:**
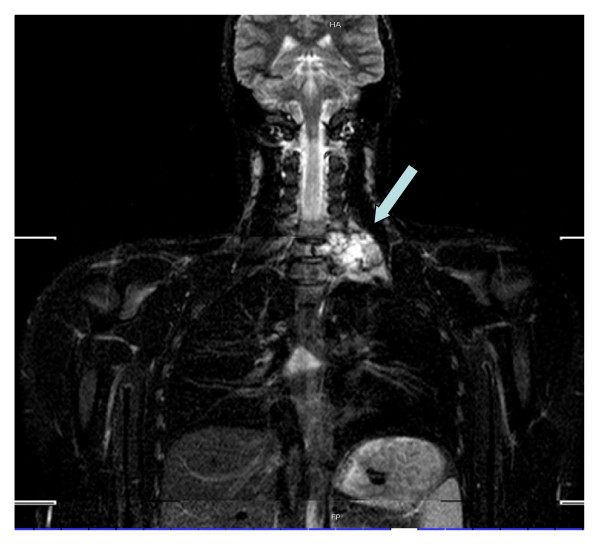
**The coronal plane from the mobi-view MRI scan shows the expansiveness of the cyst (arrow)**.

**Figure 4:**
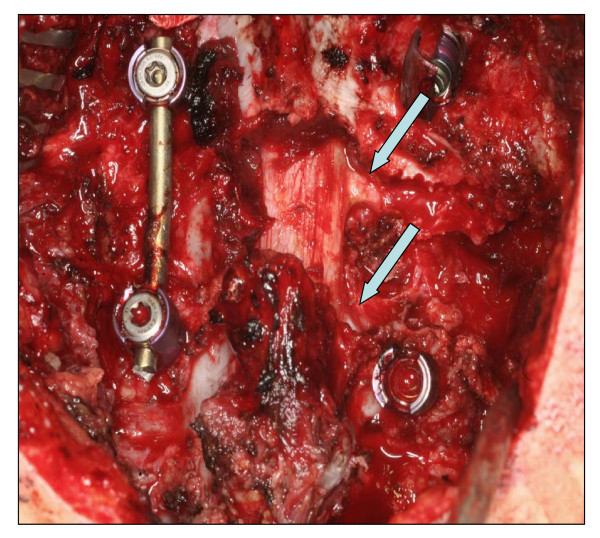
**Intraoperative view from posterior after decompression, resection of the massa lateralis und preparation of the C7 and C8 nerve roots (arrow)**.

The surgical procedure included a single-session two-step interdisciplinary approach. The first step consisted of the posterior-to-anterior intralesional resection with a subtotal spondylectomy of the 7^th ^cervical vertebra and posterior screw-and-rod fixation (Figure 3 and 4). The second step included the tricortical bone grafting (iliac crest harvesting) and anterior plating from C6 to Th1. Due to the critical envelopment of the spinal nerves a left-sided intralesional preparation from posterior was inevitable. The anterior preparation into the upper thoracic aperture was performed in collaboration with thoracic surgery. The total operating time was nine hours with a total blood loss of 2300 ml, of which 980 ml were re-transfused via cell-saver. No intraoperative complications were noted.

Postoperatively the patient showed a leakage of spinal fluid, which required temporary drainage. The cervical spine was immobilized in a semi-rigid cervical collar for three months. Sensory and motor impairment of the left arm improved instantaneously. After adequate wound healing the patient received megavoltage radiotherapy with a total dose of 30Gy, divided into single-doses of 1.8Gy throughout a three week period.

**Figure 5:**
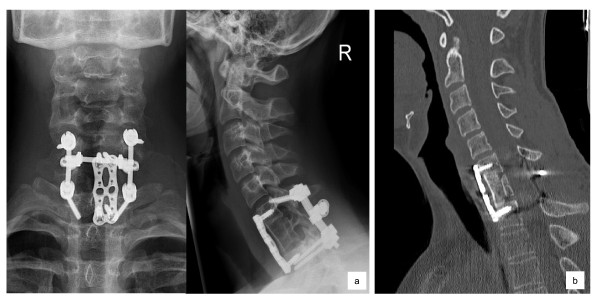
**a) The conventional X-Rays at 12 months follow-up show the correct alignment of the cervical spine with the intact bone graft and spondylodesis implants**. b) The computertomography at 12 months follow-up shows good bony consolidation and the unaltered and correct alignment of the cervical spine.

In the clinical follow-up at three, six and twelve months postoperatively, the patient showed a complete recovery of neurological functions. CT scan and conventional radiographs of the cervical spine demonstrated the intact placement of all metal implants, a good bony consolidation of the bone graft and no signs of local tumour recurrence (Figure 5).

## Discussion

Aneurysmatic bone cysts are a rare entity in the cervical spine. Although they are predominantly described to be benign bone lesions, stage 3 lesions (according to the Enneking classification) demonstrate an expansive growth and destructive behaviour with a high rate of recurrence. Neurologic impairment and pain may be a clinical prodrome for the manifestation within the spinal column. In conventional radiographs ABC appear as expansive osteolytic bone lesions with a round-oval and polycystic structure, delineated from the surrounding tissue by a thin radiopaque layer. The three-dimensional expansion of the cyst is best evaluated by CT scan. The typical CT images outline a fluid-filled cavern, often confirmed and more precisely detected on MRI, hence with improved sensitivity for the diverse fluid layering [6].

The differential diagnosis of the ABC includes the giant cell tumour, hemangioma, fibrotic dysplasia, the osteosarcoma and spinal metastasis of any other distant tumour.

The primary goal in the therapeutic regimen of spinal ABC is to completely resect, decompress neurologic and vascular structures and to re-establish and secure biomechanical stability. However, these goals may provoke ambivalent thinking when the sufficient degree of surgery for an otherwise benign tumour and the protection of sensitive neurologic structures and their anatomical surrounding are considered.

Cervical spine ABC's often involve the posterior column, which then require posterior decompression and/or resection in terms of a laminectomy, respectively. In order to prevent postlaminectomy kyphosis, additive fusion is highly recommended [7].

The low incidence of cervical spine ABC, the small and inhomogeneous number of reports and the absence of applying any form of classification make a comparison among different surgical techniques and treatment concepts difficult. Moreover, most studies involve long follow-up periods and therefore bear the risk of changing therapeutic modalities within the study itself.

Therefore, with regard to the case reported here, we aim to discuss the different modalities prophylaxis of tumour recurrence, even in cases where a complete resection is not feasible due to the expansion of the bone lesion.

Recent studies have reported a high incidence of tumour recurrence in cases of incomplete primary resection, most of which appeared within the initial 6 to 12 months after surgery [5,8,9]. Perioperative approaches to minimize this risk have included the selective arterial embolisation (SAE) and the filling of the cyst cavity with PMMA cement.

ABC at the spine requires an angiography in the diagnostic set-up to analyse the vascularity and to support the decision making process regarding the proper therapeutic approach. As mentioned above, embolization as alternative treatment option has to be taken into account. This procedure has shown to be successful in case of ABC located at the extremities. There are cases described in the literature, where embolization alone was able to heal SAE localized at the spine without further complications [1]. If ABC is located at the cervical spine, care has to be taken regarding embolization. Angiography can show embolization contraindicated, because of high risk of spinal cord damage caused by ischemia. Thus, if feasible, this procedure usually serves as a preoperative adjunct to decrease vascularity of the tumor and to reduce intraoperative blood loss [8]. If vertebral artery seems to be involved, pre-procedural occlusion test with the patient awake can reduce the risk of neurological complications [9].

Other reports have suggested postoperative radiotherapy with a high-energy low-dose application in cases with incomplete resection [10-12]. To date, radiotherapeutic protocols favour a total dose of 26-30 Gy, which is partitioned into daily doses of 1.8-2.0 Gy [10,12]. The application of megavoltage techniques has significantly reduced the epi- and subdermal side-effects as well as the scattered radiation. However, in children and adolescents radiotherapy of regions close to the spinal cord is to be considered cautiously, if not contraindicated. Although the risk of radiation-induced neoplasia must be put into perspective due to inadequate and obsolete radiation techniques and dosages in the past, growth disturbances, secondary malignoma and the development of myelopathy have been reported [13].

Past studies have demonstrated the effective use of megavoltage radiotherapy in both a monotherapeutic or combined surgical and radiotherapeutic approach. Yavuz and colleagues [14] demonstrated the successful outcome of the surgical resection and postoperative radiotherapy with a total dose of 25 Gy in a 13 year old girl with an expansive ABC located in the sternum. Feigenberg and colleagues [15] described a monotherapeutic approach using megavoltage radiotherapy in nine patients with ABC, of which three patients had their lesions located in the cervical spine. None of the patients showed tumour recurrence or the development of secondary malignoma. Furthermore, Boriani and colleagues reported the successful radiotherapy in a case of recurrent ABC in the spine [1].

Thus, we believe that megavoltage radiotherapy for the prophylaxis of tumour recurrence in ABC is justified in cases, where the anatomical region is highly demanding, especially in case of possible ABC recurrence. As presented in our case, careful preparation of both the 7^th ^and 8^th ^spinal nerve, as well as a long section of the vertebral artery was necessary allowing only for intralesional/incomplete resection.

The decision should then be based on an interdisciplinary approach amongst others evaluating body height of both parents and the skeletal maturation of the patient to estimate possible growth disturbances. In our case, this evaluation revealed a state assuming skeletal growth completion and the postoperative megavoltage radiotherapy discussed and agreed upon as a decision by consensus of the interdisciplinary tumour conference.

## Conclusion

Postoperative megavoltage radiotherapy applied within the range of today's standardized protocols can be an effective adjuvant in the treatment of ABC of the cervical spine. Exact preoperative staging and multiple biopsies to confirm the diagnosis, along with the careful evaluation of all risk factors, should be mandatory. This also implies the need for a comprehensive and continuous follow-up of patients receiving such complex treatments. More particular, this case demonstrates the challenges of the decision-making process in the treatment strategy of stage 3 ABC to be a rather complex and interdisciplinary issue as the case arises. Therefore, each case is to be evaluated individually.

## Competing interests

The authors declare that they have no competing interests.

## Authors' contributions

JR and SKT wrote the manuscript. JG and MW collected the data pre- and postoperatively. UE and CEH operated the patient and revised the manuscript critically. CEH and GVS-S gave input regarding the design and gave the final approve of the version to be published. All authors read and approved the manuscript.

## Consent statement

Written informed consent was obtained from the patient for publication of this case report and accompanying images. A copy of the written consent is available for review by the Editor-in-Chief of this journal.
